# Heat shock proteins: a therapeutic target worth to consider

**DOI:** 10.14202/vetworld.2015.46-51

**Published:** 2015-01-13

**Authors:** Amita Dubey, K. S. Prajapati, Madhu Swamy, V. Pachauri

**Affiliations:** 1 Department of Pathology, College of veterinary science & AH, NDVSU, Jabalpur, Madhya Pradesh, India; 2 Department of Pathology, College of veterinary science & AH, AAU, Anand, Gujarat, India; 3 Krishi Vigyan Kendra, Jawaharlal Nehru Agricultural University, Sagar, Madhya Pradesh, India

**Keywords:** heat shock protein, heat shock protein 70, heat shock protein 90, stress protein, small heat shock proteins

## Abstract

Heat shock proteins (HSPs) are the molecular chaperones, that are not only expressed during the normal growth process of cell cycle consecutively, but also get induced in cells during various stress conditions produced by cellular insult, environmental changes, temperature, infections, tumors etc. According to their molecular weight and functions, HSPs are divided into five major families. HSP90, HSP70, HSP60 and HSP100 are the most studied members of the family. Experimental studies have proved that overexpression and/or inhibition of HSPs play an important role in maintaining the tolerance and cell viability under above-described stress conditions. HSP90 is found to be a promising the candidate for the diagnosis, prognosis and treatment of cancer. Similarly, HSP70, HSP60 and small HSPs experimentally and clinically have potential for the treatment of neurodegenerative disease, ischemia, cell death, autoimmunity, graft rejection, etc. In a way, exploring, the cytoprotective and immunoregulatory role of HSPs can open a new avenue for the drug discovery and treatment of critical diseases.

## Introduction

The heat shock response was first described in 1962 by Ritossa and is named as heat shock proteins (HSPs) based on their increased synthesis after heat shock in house fly [1]. Later it has been noted that HSPs exist in all the organisms from bacteria to humans, and they are among the most conserved proteins known [2]. HSPs, are multimolecular complexes expressed constitutively (up 5-10% of the total protein) under normal growth condition in cells and act as molecular chaperones, which play a regulatory role in the folding of proteins, intracellular transport of proteins in cytosol, endoplasmic reticulum and mitochondria; repair or degradation of proteins and refolding of misfolded proteins [3]. In addition to being constitutively expressed, these proteins are markedly induced (up to 15%) by a range of environmental, pathological, or physiological stimuli [4]. These proteins are also modulated by nutrient deprivation, oxidative stress, hypoxia-ischemia, apoptotic stimuli and neuronal injury in the brain, etc. [5-7]. HSPs are divided into five major families, HSP100, 90, 70, 60, and the small HSP (sHSP)/α-crystallins, according to their molecular weight, structure and function. HSP synthesis results in tolerance to insult, such as thermotolerance or stress tolerance in various organisms [8]. In various acute and chronic cell injuries, pathogenic conditions such as malignancies, and infectious diseases overexpression of HSPs were found to be playing cytoprotective and immunoregulatory roles [9]. Recently, HSP reactivity in autoimmune diseases and transplantation have been proven to be down-regulated in the disease process [10,11]. Togetherness, induction or inhibition of HSPs provides vast area of therapeutic target for combating various diseases. Considering, the regulatory role of HSPs in physiological and pathological conditions, HSPs have emerged as potential drug candidates for drug development and can be a breakthrough in the near future.

## Regulation of HSPs

**Figure-1:**
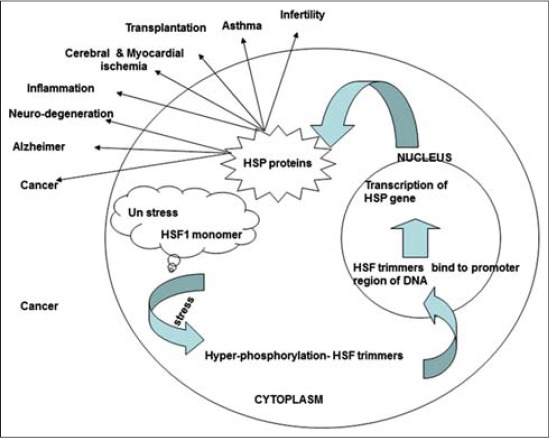
Regulation and role of heat shock proteins.

Stress condition causes protein unfolding, misfolding or aggregation, which triggers the stress response that leads to the induction of gene transcription of proteins. HSP gene transcription is mediated by the interaction of the heat shock factor (HSF1) with heat shock elements (HSEs) in the HSP gene promoter regions. In unstressed state, HSF1 is present in the cytoplasm as a latent monomeric molecule (Figure-1). Under stress, HSF1 is hyperphosphorylated in a *ras*-dependent manner by members of the mitogen-activated protein kinase (MAPK) subfamilies (e.g. ERK1, JNK/SAPK, p38 protein kinase). HSF1 is then converted to phosphorylated trimers with the capacity to bind DNA and translocates from the cytoplasm to the nucleus [12,13]. The generation of HSPs is transient, and the presence of HSPs negatively influences the protein homeostasis. The activity of HSF trimers is downregulated by HSPs (e.g. HSP70) and the heat shock binding protein 1 which is found in the nucleus [9,14].

## HSP90- A Potential Target for Cancer Therapy

HSP90 is one of the most studied and abundant proteins of eukaryotic cells, comprising 1-2% of total proteins under non-stress conditions. It is evolutionarily conserved among species and is essential for cell survival. HSP90 along with their co-chaperones, play an important role in the folding of at least 200 specific proteins of various signaling pathways, and in the refolding of denatured proteins after stress [15,16]. HSP90 interacts with and stabilizes a growing list of various kinases including several key members of malignant transformation, such as the ErbB2, Src, Abl or Met tyrosine kinases, or the Raf, Akt and cyclin-dependent serine kinases. Besides these, HSP90 is necessary for the maturation of several transcription factors, like the nuclear hormone receptors and the hypoxia-inducible factor-1. HSP 90 client proteins have been shown to be important for the development, proliferation and survival of several types of cancer. Hence inhibiting the activities of HSP90 has anti-cancer potential, N-terminal domain contains a rather unique ATP-binding site, the Bergerat-fold and more recently discovered C-terminal domain, opened the new possibilities for the inhibition of this chaperone to target the tumor [17,18]. In addition, HSP90 binding has been shown to contribute to the accumulation of tumor suppressor transcription factor p53 [19,20]. 17-AAG (17-allylamino-17-demethoxygeldanamycin), Tanespimycin, Ganetespib are examples of chemical entity inhibiting the actions of HSP 90, emerging as a potential candidate for the treatment of cancer like multiple myeloma, lung cancer, adenocarcinoma, pheochromocytoma, lymphoblastic leukemia [21-24].

## HSP70 - A Potential Target for Neuro-degenerative Diseases

Neurodegenerative diseases are great challenges to the medical field. Effective therapies are rare and applied after a vast amount of neurons is lost. Examples of neurodegenerative diseases are Alzheimer‘s disease, Parkinson’s disease, Spinal and bulbar muscular atrophy, polyglutamine diseases, Huntington’s disease (HD) and several forms of spinocerebellar ataxia, etc. A common pathogenesis of neurodegenerative disease is the misfolding, post-translation and aggregation of specific neuronal proteins [25]. A striking finding noted in various *in vitro* and *in vivo* animal experiments, are the expression of HSPs HSP70 and HSP27 in surviving neuronal cells [26-28]. They were found to be protecting the neurons against a variety of adverse conditions including oxidative stress, alongwith anti-apoptotic activity by blocking the function of several key pro-apoptotic factors. Overexpression of HSP70 in Drosophila model of Parkinson disease and 5XFAD mouse model of Alzheimer disease delayed the progression of disease by autophagy of misfolded proteins and reduction of caspase-3 induced cell death [6,29,30]. Experiments have shown protection of brain striatal lesion in HSP-70 overexpressing mice that developed the HD by Malonate and 3-nitropropionic acid [7]. Similarly in lysosomal diseases, severe brain damage occurs due to unstable mutant enzyme proteins leading to rapid intracellular degradation and loss of neuronal function. A chemical inhibitor of HSP70 has been reported to stabilize the enzyme activities [31,32].

## HSP70 in Cerebral and Myocardial Ischemia

Increased expression of HSP70 is noted in ischemic pneumbra indicating its important role in attenuation of progression and protection of ischemia. Researchers have shown increased expression of inducible HSP70 in hypoxic neuron cultures during thermal stress, global and focal cerebral ischemia [33,34]. Cerebral ischemia models using HSP70 overexpressing transgenic mice were protected against myocardial ischemia and brain ischemia (middle cerebral artery occlusion) [35]. Constitutive overexpression of HSP70 in a cytomegalovirus enhancer combined with a bactin promoter resulted in complete recovery of infarct size [36]. Salvianolate, a chemical entity, unregulated the HSP22 and protected the rat from cerebral ischemia-reperfusion injury [37]. Preconditioning with ischemia or chemical or thermal stress had proved to enhance the expression of HSP in the cell and increased the tolerance of the cell [38].

## HSPs in Inflammation

Role of HSPs in induction of several pro-inflammatory cytokines and the signaling pathways are yet to be fully clarified. Though the studies have shown that overexpression of HSP60 induces the secretion of interleukin 6 (IL-6) from human monocytes via signaling through CD 14 and p38 MAPK, along with the enhanced expression of TNF-α and nitric oxide. It also induces the expression of a range of cytokines, including IL-12 and IL-15 [39-41]. Mammalian HSP60 has been proved as a key target for T cell and Ab responses in chronic inflammation and atherosclerosis [42]. Bacterial HSPs have shown to enhance the expression of intracellular cell adhesion molecule and vascular cell adhesion molecule. HSP70 acts through a CD14-dependent pathway to stimulate IL-1, IL-6 and TNF-α production, indicating the fact that HSPs are directly involved in the inflammation [41].

## HSP in Transplantation

HSPs have been associated with allograft rejection and in autoimmunity specially the HSP60. Graft-infiltrating lymphocytes proliferate in response to recombinant mycobacterial HSP65 and HSP71. Multiple HSPs (HSp27, 60 and 70) are articulated in renal allografts [10]. In rats and humans, increased expression of HSP70 gene and protein are reported in rejection of cardiac allografts. HSP expression was also induced in the small intestinal epithelium of rat after small-bowel transplantation and found to be resistant to immunosuppressive drug tacrolimus [43]. These findings have pointed out that the HSP reactivity in allograft would have a role in the acute and chronic graft rejection [44]. However, the down-regulation of HSPs in autoimmune and graft rejection process, activation of innate immunity by these proteins are also meaningful as evident by the prolonged survival of murine skin allograft at effect of HSP60 immunity. It has also be reported that overexpression of HSP27 in mouse hearts delays the allograft rejection by inhibiting the tissue damage. Indeed, the presence of HSPs and HSP specific lymphocytes are not always indicative of a deleterious response, they might reflect an anti-inflammatory protective response also [45,46].

## HSP in Reproduction

HSP production is enhanced during germ cell development and *in-vitro* embryo culture, and they are among the first proteins produced during mammalian embryo growth. The spontaneous expression of HSP as an essential part of embryo development is well documented, and the presence or absence of HSP influences various aspects of reproduction in many species [47]. Increased HSP27, HSP60 and heat shock cognate 70 in the endometrium are expressed after ovulation and in the early secretory phase, which is the critical period of endometrial receptivity for an implanting embryo [4]. Studies on *in vitro* cultured mouse and bovine embryos demonstrate that monoclonal antibodies to the 60 and 70 kDa HSPs (HSP60, HSP70) inhibit early embryo development. Genital tract infections, especially chronic Chlamydia trachomatis infections of the fallopian tubes, result in the generation of immunity to conserved regions that are present on both the chlamydial and human HSP60 molecules [48]. HSPs are consecutively expressed and have their role in all stages of spermatogenesis as evident in various studies on experimental animals and human. HSP70 knockout mice resulted in a failed meiosis, massive apoptosis, and male infertility, in another experiment HSP 90 knockout mice displayed sterility because of morphological abnormality. Similarly, disruption of sHSP, HSPB10 resulted in lower sperm motility and male infertility in a rat [49].

## SHSPs

Mammalian HSP27 (HSPB1) and άB-crystallin (HSPB5) belong to the family of sHSPs. In human, 20 different sHSPs have been characterized but only few of them, such as HSP27, HSP22 and άB-crystallin, are true HSPs. The structural organization of sHSPs appears to be a crucial factor, which controls the activity of these proteins [14]. sHSP is constitutively expressed in tissues with high rates of oxidative metabolism, including, the heart, type I and type IIa skeletal muscle fibers, brain, eye and oxidative regions of the kidney. Their expression in these organs indicates these can be therapeutic targets for neurodegenerative diseases, myopathies, asthma, cataracts and cancers [50,51]. HSP27 is involved in cell immunity, inflammation, thermoregulation, tumorogenesis, and apoptosis. HSP27 in murine L929 cell line blocks the Fas/Apo-1 mediated cell death. It also protects the actin and intermediate filaments from the fragmentation and modulates the ROS glutathione levels [52]. Similarly, HSP22 is probably involved in the cell proliferation regulation, cardiac hypertrophy, apoptosis, neuromuscular health and carcinogenesis [53]. Another member of family αB-crystallin has shown to have a protective effect on myocardial cells and retina from the oxidative injury [54-56].

## HSPs in Veterinary Research

Romanucci demonstrated that HSP27, HSP72 and HSP90 are involved in canine mammary gland carcinogenesis [54]. In addition, HSP27 appears to be implicated in tumor invasiveness and that is indicative of a poor prognosis. In veterinary literature, high levels of HSP60 and HSP70 were reported in canine transmissible venereal tumor (CTVT), and it was thought that these HSPs could be considered potential markers for CTVT cells [57-59]. More recent studies have shown high levels HSP70 expression in canine mammary tumors confirming that HSP expression cannot be relied upon for the recognition of a specific tumor histological type. Bovine leukemia virus (BLV) infection up-regulates the expression of HSPs which in turn could augment the humoral response. Anti-HSP70 activity was examined in the sera of 34 BLV-infected cattle and 40 healthy controls by ELISA. Significantly higher activities (p<0.001) were observed in BLV-infected animals [60]. Several studies have proposed the implication of HSP in prion diseases. Serrano *et al*., demonstrated that HSPs are involved in gliosis and inflammatory reactions rather than in anti-apoptotic processes in natural scrapie [61].

## Conclusion

HSPs are emerging as therapeutic targets for human as well as veterinary medicine. Though a lot has been studied in recent years, there are still many aspects of HSP that remain puzzling. On one hand, HSPs appears to be associated with several pathological conditions, on the other side these are ubiquitously expressed and have a physiological role. HSPs are implicated in all phases of cancer from proliferation, impaired apoptosis and sustained angiogenesis to invasion and metastasis. The presence of abnormal HSP levels in several human tumors suggests that these proteins could be used as diagnostic and/or prognostic markers, whilst the direct correlation between HSP expression and drug resistance in neoplastic tissues means they can also be used to predict cancer response to specific treatment. Experimental and clinical studies with HSPs showed a new opportunity for the treatment of ischemia and neurodegenerative diseases. The observation that HSPs directly or indirectly elicit potent immunoregulatory activities give a new perspective on the roles of HSPs and anti-HSP reactivity in autoimmunity, transplantation, vascular disease, and other conditions. Though the HSPs have also been successfully targeted in clinical trials, still there is scope for exploring its expression and inhibitory effects on the normal and as well as stressed cells, best mimic animal model and translation of the experimental work to the clinical condition. Similarly sHSPs are also emerging as fascinating class of proteins, might be crucial for future therapeutic strategies for critical diseases such as cancer, neurodegenration, etc., if the scientists are able to unravel developmental mechanisms, gene transcription and tissue- functions.

## Author’s Contributions

AD conceptualized and prepared initial version of the manuscript. VP helped in the designing and literature collection and reference arrangement. KSP and MS reviewed the manuscript for critical scientific correction and helped to structure the manuscript in its final shape.

## References

[1] Ritossa F (1962). A new puffing pattern induced by temperature and DNP in Drosophila. Experientia.

[2] Schlesinger M.J (1990). Heat shock proteins. J. Biol. Chem.

[3] Hendrick J.P, Hartl F.U (1993). Molecular chaperone functions of heat-shock proteins. Annu. Rev. Biochem.

[4] Millar L.N, Murrell G.A.C (2012). Heat shock proteins in tendinopathy: Novel molecular regulators. Mediators. Inflamm 2012.

[5] Bellmann K, Jaattela M, Wissing D, Burkart V, Kolb H (1996). Heat shock protein Hsp70 over expression confers resistance against nitric oxide. FEBS Lett.

[6] Calabrese V, Cornelius C, Maiolino L, Luca M, Chiaramonte R, Toscano M.A, Serra A (2010). Oxidative stress redox homeostasis and cellular stress response in Ménière's disease: Role of vitagenes. Neurochem. Res.

[7] Choi Y.J, Kim N.H, Lim M.S, Lee H.J, Kim S.S, Chun W (2014). Geldanamycin attenuates 3 - Nitropropionic acid - Induced apoptosis and JNK activation through the expression of HSP 70 in striatal cells. Int. J. Mol. Med.

[8] Parsell D.A, Lindquist S, Morimoto R.I, Tissières A, Georgopoulos C (1994). Heat shock proteins and stress tolerance. The Biology of Heat Shock Proteins Molecular Chaperones.

[9] Pathan M.M, Latif A, Das H, Siddiquee G.M, Khan J.Z (2010). Heat Shock Proteins and their clinical Implications. Vet. World.

[10] Coelho V, Faria A.M (2012). HSP60: Issues and insights on its therapeutic use as an immunoregulatory agent. Front. Immunol.

[11] O'Neill S, Ingman T.G, Wigmore S.J, Harrison E.M, Bellamy C.O (2013). Differential expression of heat shock proteins in healthy and diseased human renal allografts. Ann. Transplant.

[12] Morimoto R.I (1998). Regulation of the heat shock transcriptional response: Cross talk between a family of heat shock factors molecular chaperones and negative regulators. Gene Dev.

[13] Pratt W.B, Toft D.O (2003). Regulation of signaling protein function and trafficking by the hsp90/hsp70-based chaperone machinery. Exp. Biol. Med.

[14] De Thonel A, Le Mouël A, Mezger V (2012). Transcriptional regulation of small HSP-HSF1 and beyond. Int. J. Biochem. Cell B.

[15] Csermely P, Schnaider T, Soti C, Prohaszka Z, Nardai G (1998). The 90-kDa molecular chaperone family: Structure function and clinical applications: A comprehensive review. Pharmacol. Ther.

[16] Csermely P, Agoston V, Pongor S (2005). The efficiency of multi-target drugs: The network approach might help drug design. Trends Pharmacol. Sci.

[17] Garnier C, Lafitte D, Tsvetkov P.O, Barbier P, Leclerc-Devin J, Millot J.M, Briand C, Makarov A.A, Catelli M.G, Peyrot V (2002). Binding of ATP to heat shock protein 90: Evidence for an ATP-binding site in the C-terminal domain. J. Biol. Chem.

[18] Jego G, Hazoumé A, Seigneuric R, Garrido C (2013). Targeting heat shock proteins in cancer. Cancer Lett.

[19] Richardson P.G, Mitsiades C.S, Laubach J.P, Lonial S, Chanan-Khan A.A, Anderson K.C (2011). Inhibition of heat shock protein 90 (HSP90) as a therapeutic strategy for the treatment of myeloma and other cancers. Br. J. Haematol.

[20] Okayama S, Kopelovich L, Balmus G, Weiss R.S, Herbert B.S, Dannenberg A.J, Subbaramaiah K (2014). p53 protein regulates Hsp90 ATPase activity and thereby Wnt signaling by modulating Aha1 expression. J. Biol. Chem.

[21] Banerji U, O'donnell A, Scurr M, Pacey S, Stapleton S, Asad Y, Simmons L, Malone Y.A, Raynaud F, Campbel L.M, Walton M, Lakhani S, Kaye S, Workman P, Judson I (2005). Phase I pharmacokinetic and pharmacodynamic study of 17-allylamino 17-demethoxygeldanamycin in patients with advanced malignancies. J. Clin. Oncol.

[22] Tavernier E, Flandrin-Gresta P, Solly F, Rigollet L, Cornillon J, Augeul-Meunier K, Stephan J.L, Montmartin A, Viallet A, Guyotat D, Campos L (2012). HSP90 inhibition results in apoptosis of Philadelphia acute lymphoblastic leukaemia cells: An attractive prospect of new targeted agents. J. Cancer Res. Clin.

[23] Giubellino A, Sourbier C, Lee M.J, Scroggins B, Bullova P, Landau M, Ying W, Neckers L, Trepel J.B, Pacak K (2013). Targeting heat shock protein 90 for the treatment of malignant pheochromocytoma. PLoS One.

[24] Tosti G, Cocorocchio E, Pennacchioli E, Ferrucci P.F, Testori A, Martinoli C (2014). Heat-shock proteins-based immunotherapy for advanced melanoma in the era of target therapies and immunomodulating agents. Expert Opin. Biol. Ther.

[25] Paul S, Mahanta S (2014). Association of heat-shock proteins in various neurodegenerative disorders: Is it a master key to open the therapeutic door?. Mol. Cell. Biochem.

[26] Di Domenico F, Sultana R, Tiu G.F, Scheff N.N, Perluigi M, Cini C, Butterfield D.A (2010). Protein levels of heat shock proteins 27, 32, 60, 70, 90 and thioredoxin-1 in amnestic mild cognitive impairment: An investigation on the role of cellular stress response in the progression of Alzheimer disease. Brain Res.

[27] Cornelius C, Trovato Salinaro A, Scuto M, Fronte V, Cambria M.T, Pennisi M, Bella R, Milone P, Graziano A, Crupi R, Cuzzocrea S, Pennisi G, Calabrese V (2013). Cellular stress response sirtuins and UCP proteins in Alzheimer disease: Role of vitagenes. Immun. Ageing.

[28] Wang X, Cattaneo F, Ryno L, Hulleman J, Reixach N, Buxbaum J.N (2014). The systemic amyloid precursor transthyretin (TTR) behaves as a neuronal stress protein regulated by HSF1 in SH-SY5Y human neuroblastoma cells and APP23 Alzheimer's disease model mice. J. Neurosci.

[29] Ebrahimi-Fakhari D, Saidi L.J, Wahlster L (2013). Molecular chaperones and protein folding as therapeutic targets in Parkinson's disease and other synucleinopathies. Acta Neuropathol. Commun.

[30] Bobkova N.V, Garbuz D.G, Nesterova I, Medvinskaya N, Samokhin A, Alexandrova I, Yashin V, Karpov V, Kukharsky M.S, Ninkina N.N, Smirnov A.A, Nudler E, Evgenev M (2014). Therapeutic effect of exogenous hsp70 in mouse models of Alzheimer's disease. J. Alzheimers Dis.

[31] Suzuki Y, Ogawa S, Sakakibara Y (2009). Chaperone therapy for neuronopathic lysosomal diseases: Competitive inhibitors as chemical chaperones for enhancement of mutant enzyme activities. Perspect. Med. Chem.

[32] Shukla A.K, Pragya P, Chaouhan H.S, Tiwari A.K, Patel D.K, Abdin M.Z, Chowdhuri D.K (2014). Heat shock protein-70 (Hsp-70) suppresses paraquat-induced neurodegeneration by inhibiting JNK and caspase-3 activation in drosophila model of Parkinson's disease. PLoS One.

[33] Yenari M.A, Giffard R.G, Sapolsky R.M, Steinberg G.K (1999). The neuroprotective potential of heat shock protein 70 (HSP70). Mol. Med. Today.

[34] Sharp F.R, Zhan X, Liu D.Z (2013). Heat shock proteins in the brain: Role of Hsp70 Hsp 27 and HO-1 (Hsp32) and their therapeutic potential. Transl. Stroke Res.

[35] Marber M.S, Mestril R, Chi S.H (1995). Overexpression of the rat inducible 70-kD heat stress protein in a transgenic mouse increases the resistance of the heart to ischemic injury. J. Clin. Invest.

[36] Rajdev S, Hara K, Kokubo Y, Mestril R, Dillmann W, Weinstein P.R, Sharp F.R (2000). Mice overexpressing rat heat shock protein 70 are protected against cerebral infarction. Ann. Neurol.

[37] Zhang J, Lu W, Lei O, Tao X, You H, Xie P (2013). Salvianolate increases heat shock protein expression in a cerebral ischemia-reperfusion injury model. Neural Regen. Res.

[38] Xia D.Y, Li W, Qian H.R, Yao S, Liu J.G, Qi X.K (2013). Ischemia preconditioning is neuroprotective in a rat cerebral ischemic injury model through autophagy activation and apoptosis inhibition. Braz. J. Med. Biol. Res.

[39] Galdiero M, Del'Ero G.C, Marcatili A (1997). Cytokine and adhesion molecule expression in human monocytes and endothelial cells stimulated with bacterial heat shock proteins. Infect. Immun.

[40] Yadav A.K, Kumar V, Jha V (2013). Heat shock proteins 60 and 70 specific proinflammatory and cytotoxic response of CD4+CD28 null cells in chronic kidney disease. Mediators Inflamm 2013.

[41] Lovett M.C, Coates J.R, Shu Y, Oglesbee M.J, Fenner W, Moore S.A (2014). Quantitative assessment of hsp70 IL-1βand TNF-αin the spinal cord of dogs with E40K SOD1-associated degenerative myelopathy. Vet. J.

[42] Grundtman C, Kreutmayer S.B, Almanzar G, Wick M.C, Wick G (2011). Heat shock protein 60 and immune inflammatory responses in atherosclerosis. Arterioscler. Thromb. Vasc. Biol.

[43] Wang J, Li Y, Li J (2013). Cell stress response in rat chronic small bowel allograft rejection. Transplant. Proc.

[44] Van Eden W, Bonorino C, Van Der Zee R (2013). The immunology of cellular stress proteins. Front. Immunol.

[45] Pockley A.G, Muthana M (2005). Heat shock proteins and allograft rejection. Contrib. Nephrol.

[46] Seemampillai B, Germack R, Felkin L.E, McCormack A, Rose M.L (2014). Heat shock protein-27 delays acute rejection after cardiac transplantation: An experimental model. Transplantation.

[47] Neuer A, Spandorfer S.D, Giraldo P, Dieterle S, Rosenwaks Z, Witkin S.S (2000). The role of heat shock proteins in reproduction. Hum. Reprod. Update.

[48] Linhares I.M, Witkin S.S (2010). Immunopathogenic consequences of Chlamydia trachomatis 60 kDa heat shock protein expression in the female reproductive tract. Cell Stress Chaperone.

[49] Ji Z, Duan Y, Mou L, Allam J, Haidl G, Cai Z (2012). Association of heat shock proteins, heat shock factors and male infertility. Asian Pac. J. Reprod.

[50] Acunzo J, Katsogiannou M, Rocchi P (2012). Small heat shock proteins HSP27 (HspB1), αB-crystallin (HspB5) and HSP22 (HspB8) as regulators of cell death. Int. J. Biochem. Cell B.

[51] Reddy P.S, Kavi Kishor P.B, Seiler C, Kuhlmann M, Eschen-Lippold L, Lee J, Reddy M.K, Sreenivasulu N (2014). Unraveling regulation of the small heat shock proteins by the heat shock factor HvHsfB2c in barley: Its implications in drought stress response and seed development. PLoS One.

[52] Wood K.L, Nunley D.R, Moffatt-Bruce S, Pope-Harman A, Huang Q, Shamo E.N, Phillips G.S, Baran C, Batra S, Marsh C.B, Doseff A.I (2010). The role of heat shock protein 27 in bronchiolitis obliterans syndrome after lung transplantation. J. Heart Lung Transpl.

[53] Shemetov A.A, Seit-Nebi A.S, Gusev N.B (2008). Structure, properties, and functions of the human small heat-shock protein HSP22 (HspB8, H11, E2IG1): A critical review. J. Neurosci. Res.

[54] Tang S, Lv Y, Chen H, Adam A, Cheng Y, Hartung J, Bao E (2014). Comparative analysis of αB-crystallin expression in heat-stressed myocardial cells *in vivo* and *in vitro*. PLoS One.

[55] Xu F, Yu H, Liu J, Cheng L (2013). αB-crystallin regulates oxidative stress-induced apoptosis in cardiac H9c2 cells via the PI3K/AKT pathway. Mol. Biol. Rep.

[56] Parfitt D.A, Aguila M, McCulley C.H, Bevilacqua D, Mendes H.F, Athanasiou D, Novoselov S.S, Kanuga N, Munro P.M, Coffey P.J, Kalmar B, Greensmith L, Cheetham M.E (2014). The heat-shock response co-inducer arimoclomol protects against retinal degeneration in rhodopsin retinitis pigmentosa. Cell Death Dis.

[57] Romanucci M, Marinelli1 A, Sarli G, Salda L.G (2006). Heat shock protein expression in canine malignant mammary tumors. BMC Cancer.

[58] Chu R.M, Sun T.J, Yang H.Y, Wang D.G, Liao K.W, Chuang T.F, Li C.H, Lee W.C (2001). Heat shock proteins in canine transmissible venereal tumor. Vet. Immunol. Immunopathol.

[59] Selvarajah G.T, Bonestroo F.A, Kirpensteijn J, Kik M.J, Van der Zee R, Van Eden W, Timmermans-Sprang E.P, Slob A, Mol J.A (2013). Heat shock protein expression analysis in canine osteosarcoma reveals HSP60 as a potentially relevant therapeutic target. Cell Stress Chaperon.

[60] Unger-Waron H, Brenner J, Paz R, Moalem U, Trainin Z (1996). gamma delta T-lymphocytes and anti-heat shock protein reactivity in bovine leukemia virus infected cattle. Vet. Immunol. Immunopathol.

[61] Serrano C, Bolea R, Lyahyai J, Filali H, Varona L, Marcos-Carcavilla A, Cristina A, Calvo J.H, Serrano M, Badiola J.J, Zaragoza P, Martín-Burriel I (2011). Changes in HSP gene and protein expression in natural scrapie with brain damage. Vet. Res.

